# Elevated Plasma Angiopoietin-like 4 Protein Levels in Adult Patients with Dengue

**DOI:** 10.3390/v17020226

**Published:** 2025-02-06

**Authors:** Win Khaing, Suk Hiang Lau, Tun-Linn Thein, Nguan Soon Tan, Sylvie Alonso, Shawn Vasoo, Po Ying Chia, David Chien Boon Lye, Yee Sin Leo, Vincent T. K. Chow

**Affiliations:** 1 National Centre for Infectious Diseases, Singapore 308442, Singapore; win_khaing@ncid.sg (W.K.); linn_thein_tun@ncid.sg (T.-L.T.); shawn_vasoo@ttsh.com.sg (S.V.); poying_chia@ncid.sg (P.Y.C.); david_lye@ncid.sg (D.C.B.L.); 2 Infectious Diseases Translational Research Program, Department of Microbiology and Immunology, Yong Loo Lin School of Medicine, National University of Singapore, Singapore 117545, Singapore; miclsh@nus.edu.sg (S.H.L.); micas@nus.edu.sg (S.A.); 3 Lee Kong Chian School of Medicine, Nanyang Technological University, Singapore 308232, Singapore; nstan@ntu.edu.sg; 4 Department of Infectious Diseases, Tan Tock Seng Hospital, Singapore 308433, Singapore; 5 Yong Loo Lin School of Medicine, National University of Singapore, Singapore 119228, Singapore; 6 Saw Swee Hock School of Public Health, National University of Singapore, Singapore 117549, Singapore

**Keywords:** dengue, angiopoietin-like 4, endothelial dysfunction, plasma leakage, disease severity, healthy controls

## Abstract

Dengue virus infection can cause severe complications due to vascular leakage. Angiopoietin-like protein 4 (ANGPTL4) regulates vascular permeability, but its role in dengue pathogenesis is unclear. This study investigated the association between plasma ANGPTL4 levels and dengue severity in Singapore adults. Plasma samples from 48 dengue patients (24 severe and 24 non-severe) during acute and convalescent phases were selected from the prospective COhort study on progression of DENgue severity in Singapore adults (CODEN) cohort. The CODEN was conducted at the National Centre for Infectious Diseases, Tan Tock Seng Hospital, from June 2016 to January 2020. ANGPTL4 levels were measured and compared to 152 healthy controls. Logistic regression assessed the relationship between plasma ANGPTL4 concentrations and disease severity. There were no statistically significant differences in ANGPTL4 levels between severe and non-severe dengue patients during acute (677.4 vs. 909.1 pg/mL, *p* = 0.4) or convalescent phases (793.7 vs. 565.6 pg/mL, *p* = 0.96). Plasma ANGPTL4 levels were significantly elevated during acute dengue (4634.3 pg/mL) versus healthy controls (907.4 pg/mL), declining during convalescence. Compared to the lowest tertile, the adjusted odds ratios for severe dengue were 0.36 (95%CI: 0.08–1.65, *p* = 0.190) for medium tertile and 0.57 (95%CI: 0.13–2.49, *p* = 0.456) for high tertile. Among patients with high ANGPTL4 levels (>5000 pg/mL), 36.4% developed severe complications, including significant plasma leakage. Plasma ANGPTL4 levels were significantly higher in dengue patients than controls, suggesting its potential as a biomarker, which warrants future detailed investigations. Larger prospective studies with serial sampling, including pediatric populations, may clarify the role of ANGPTL4 in severe dengue.

## 1. Introduction

Dengue is a mosquito-borne viral infection caused by the dengue virus (DENV), a member of the *Flaviviridae* family, primarily transmitted by *Aedes aegypti* mosquitoes. The global burden of dengue has significantly increased over the past decades, with rising age-stratified incidence, disability-adjusted life years, and deaths [1]. About 390 million annual infections occur worldwide, of which 25% result in symptomatic dengue infections [2]. While most are asymptomatic infections or mild illness, some patients develop severe complications such as dengue hemorrhagic fever (DHF), shock, severe bleeding, and organ dysfunction [3]. Individuals with comorbidities such as diabetes, obesity, hypertension, and asthma are at higher risk of severe dengue, marked by endothelial dysfunction [4,5]. The increases in vascular permeability and plasma leakage may potentially culminate in life-threatening complications [6,7].

Angiopoietin-like 4 (ANGPTL4) is a secreted glycoprotein belonging to the angiopoietin-like protein family and is involved in multiple biological functions such as the regulation of lipid and glucose metabolism, hematopoietic stem cell expansion, angiogenesis, and vascular functions, including inflammation and vascular permeability [8,9,10,11,12]. ANGPTL4 can be cleaved into an N-terminal fragment (N-ANGPTL4) affecting lipid metabolism and a C-terminal fragment (C-ANGPTL4) influencing vascular function [13]. ANGPTL4 exhibits both pro- and anti-inflammatory properties, depending on the context and tissue involved [14]. In vascular leakage, C-ANGPTL4 can disrupt endothelial cell integrity via interactions with cellular components and inflammatory signaling pathways, leading to increased vascular permeability [15].

ANGPTL4 is a crucial biomarker in infectious diseases characterized by endothelial dysfunction and vascular permeability. In acute respiratory distress syndrome [16], COVID-19 [17], and Kawasaki disease [18], elevated ANGPTL4 levels correlate with disease severity and poor clinical outcomes. The involvement of ANGPTL4 in vascular leakage highlights its potential as a diagnostic marker and even therapeutic target. However, the precise mechanisms by which ANGPTL4 modulates endothelial integrity and how it can be targeted therapeutically remain incompletely understood.

The interplay between DENV, endothelial dysfunction, and ANGPTL4 has gained attention recently. ANGPTL4 actively contributes to endothelial pathogenesis, directly modulating critical functions by binding to VE-cadherin and claudin-5, regulating vascular permeability and angiogenic processes [15]; it is not just a consequence of endothelial dysfunction [12]. Studies on lung infection and inflammation reveal that ANGPTL4 enhances pulmonary tissue leakiness and damage [19]. In murine influenza and pneumonia models, elevated ANGPTL4 is associated with increased vascular permeability [20]. These findings suggest that ANGPTL4 could play an essential role in the pathogenesis of vascular leakage in certain infections.

Despite the evidence from animal studies, the role of ANGPTL4 in human infections associated with vascular leakage remains relatively unexplored. Given its potential importance in vascular dysfunction and the critical role of vascular leakage in severe dengue, it is essential to investigate the relationship between ANGPTL4 and dengue severity in humans. There are no specific dengue antiviral drugs, and patient management relies on careful monitoring, supportive care, and precise fluid management. Understanding the role of ANGPTL4 in dengue pathogenesis may offer new insights into disease mechanisms and potentially novel management strategies.

The objective of this study was to measure the levels of ANGPTL4 in blood samples from dengue patients compared to healthy controls and to explore its involvement in dengue virus infection. By examining the relationship between ANGPTL4 levels and clinical disease severity, we sought to enhance our understanding of dengue pathogenesis and contribute to developing improved diagnostic and therapeutic approaches for this significant disease of global concern.

## 2. Materials and Methods

### 2.1. Ethics Statement

The dengue case cohort data were obtained with informed consent. Patients were informed about using their anonymized residual samples from admission to discharge for inpatients, and until the follow-up end for outpatients. The study protocols followed the Declaration of Helsinki (1989) and were approved by the Singapore National Healthcare Group Domain-Specific Review Board (reference number 2016/00076). Healthy control subject data were obtained with informed consent, and the study protocol was approved by the National University of Singapore Institutional Review Board (reference number B-16-051).

### 2.2. Cohort Description

The prospective COhort study on progression of DENgue severity in Singapore adults (CODEN) was conducted at the National Centre for Infectious Diseases (NCID), Communicable Disease Centre (CDC), Tan Tock Seng Hospital (TTSH), in Singapore from June 2016 to January 2020. It recruited adult patients (aged above 21 years) with a laboratory-confirmed dengue infection, defined as (a) those fulfilling WHO 1997 or 2009 clinical criteria [21,22] and being dengue IgM or IgG positive or both; or (b) testing positive for the dengue NS1 antigen; or (c) being dengue RT-PCR positive. After informed consent, daily residual blood samples were collected from the clinical diagnostic laboratory following routine testing. Data were collected by reviewing the medical records to understand disease progression, identify outcome factors, validate severe dengue biomarkers, and establish a bio-data repository. The cohort comprised 550 patients (299 males and 251 females) with a mean age of 45 and standard deviation (SD) of 18.52 years. In terms of clinical outcomes within this cohort, 66 (12%) had dengue hemorrhagic fever, 17 (3%) experienced dengue shock syndrome, 365 (66%) displayed warning signs, and 73 (13%) had severe dengue. Plasma samples were also obtained from 152 healthy control subjects who were recruited in a cohort with an age range from 38 to 60 years and a male/female ratio of 40:60.

### 2.3. Study Design and Patient Data Collection

Plasma samples from the CODEN and healthy controls were stored at −80 °C. Twenty-four severe dengue cases were selected via convenience sampling, and non-severe dengue cases were included in a 1:1 ratio based on the availability of acute and convalescent samples. All samples were blinded to the laboratory team to ensure unbiased analysis. Patients’ demographic data, medical history, comorbidities, symptoms, fever duration, complications, dengue diagnosis, laboratory results, serology, imaging, treatment details, and outcomes were collected via medical record reviews.

### 2.4. Operational Definitions

Severe dengue was defined according to the WHO 2009 criteria [22], i.e., the presence of plasma leakage leading to shock (or) fluid accumulation with respiratory distress (or) severe bleeding (or) an altered level of consciousness (or) severe gastrointestinal involvement (or) severe organ impairment. Patients who did not meet the severe dengue definition were all classified as non-severe dengue. Severe bleeding was defined as hematemesis, melena, menorrhagia, or a reduction in hemoglobin requiring a transfusion of blood products. Severe organ involvement included hepatic injury (alanine aminotransferase > 1000 U/L or aspartate aminotransferase > 1000 U/L), impaired consciousness, (or) myocarditis. Severe plasma leakage was defined as either clinical fluid accumulation or a hematocrit change of >20% in combination with at least one of the following: tachycardia (pulse > 100/min), hypotension (systolic BP < 90 mmHg), or narrow pulse pressure (<20 mmHg). In this study, the severity of plasma leakage was categorized as significant, mild, or none [23]. Significant plasma leakage was defined by a hematocrit change of >20% in the complete blood count or by the attending physician’s clinical detection of free fluid in the lungs or abdomen. Patients with a low serum total protein level (<63 g/L; normal range, 64–83 g/L) without apparent fluid accumulation were classified as having mild plasma leakage. Patients who did not fulfill these criteria were classified as having no plasma leakage. The study defined “acute” samples as those collected within the first 5 days of fever, while “convalescent” samples were collected on or after 10 fever days, with an interval of 10 to 14 days between the acute and convalescent samples.

### 2.5. Determination of Plasma ANGPLT4 Concentrations

ANGPTL4 concentrations in plasma samples were quantified at room temperature using the human angiopoietin-like 4 (ARP4) enzyme-linked immunosorbent assay (ELISA) kit (Abcam, ab99974, Cambridge, UK) according to the manufacturer’s instructions with minor modifications. Briefly, plasma samples were diluted with diluent A and assayed as two or more replicates. To plot the standard curve, the stock standard was serially diluted with diluent A to prepare standards with ANGPTL4 concentrations of 27.4, 82.3, 246.9, 740.7, 2222, 6666, and 20,000 pg/mL. Diluted samples and standards (100 µL each) were added into the wells and incubated for 2.5 h with gentle shaking. After discarding the contents, the wells were washed 4 times with a 1× wash solution before adding a biotinylated anti-human ANGPTL4 detection antibody and incubated for 1 h. After discarding the contents, the wells were washed 4 times before adding streptavidin conjugated with horseradish peroxidase and incubated for 45 min. After discarding the contents, the wells were washed 4 times before adding the TMB (3,3′,5,5′-tetramethylbenzidine) substrate reagent and incubated for 30 min in the dark. A stop solution was then added to the wells to generate a yellow color of varying intensity, and the absorbance was immediately measured at 450 nm using a Tecan Spark 10M microplate reader. The standard curve was plotted based on the standard concentrations (*x*-axis) and their optical density readings (*y*-axis). The ANGPTL4 concentration in each plasma sample was extrapolated from the standard curve based on the absorbance.

### 2.6. Statistical Analysis

Statistical analysis was performed using R version 4.4.1 [24]. Categorical variables were summarized as frequencies and percentages, while continuous variables were summarized as means, standard deviations, medians, and interquartile ranges. The chi-squared, Fisher’s exact tests, Mann–Whitney U test (MWU) for independent samples, and the Wilcoxon signed-rank (WSR) test for paired samples were employed. ANGPTL4 protein concentrations were transformed into log2 and stratified into tertiles. We used logistic regression with generalized linear models and the binomial family (“glm” in the R “stats” package v3.6.2) to test for the association between ANGPTL4 concentration and dengue severity, presenting as the adjusted odds ratio (aOR) with confidence intervals (CIs) and *p*-values [24]. All regression analyses were controlled for age, gender, plasma leakage, and comorbidity.

## 3. Results

### 3.1. Characteristics of Selected Dengue Patients

The clinical and laboratory characteristics of the selected dengue patients are shown in Table 1. Among 48 patients with dengue infection (24 severe dengue and 24 non-severe dengue, all with dengue NS1-positive results), this cohort’s mean age was 45.7 years (SD 15.4). The cohort comprised 25 males (52.08%) and 23 females (47.92%), with Chinese ethnicity predominating (79.17%). Most patients (83.33%) required hospitalization, and almost one-fifth (18.75%) had comorbidities. Dengue hemorrhagic fever (DHF) was diagnosed in 10 patients (20.83%). Plasma leakage was observed in varying degrees: 33.33% had significant leakage, 10.42% had mild leakage, and 56.25% had no leakage. In comparing severe dengue and non-severe dengue cases, severe cases tended to be older (48.8 vs. 42.7 years, *p* = 0.2) and were more likely to be female patients (62.50% vs. 37.50%, *p* = 0.043). All severe cases required hospitalization compared to 66.67% of non-severe cases (*p* = 0.004). DHF was more prevalent in the severe dengue group (33.33% vs. 8.33%, *p* = 0.033). Significant plasma leakage was observed in 54.17% of severe cases compared to 12.50% of non-severe cases (*p* = 0.005).

### 3.2. Plasma ANGPTL4 Levels in Dengue Patients at Acute and Convalescent Phases

ANGPTL4 levels were measured in the acute and convalescent phases of dengue (Table 2). In the acute phase, the median level was 870.6 pg/mL (IQR: 441.7 to 4368.4 pg/mL), ranging from 175.2 to 63,737.6 pg/mL. Median acute levels were lower in severe dengue cases (677.4 vs. 909.1 pg/mL, MWU test *p* = 0.4). In the convalescent phase, the median level was 581.5 pg/mL (IQR 315.3 to 2191.8 pg/mL), ranging from 92.3 to 19,074.2 pg/mL. Median convalescent levels were higher in severe cases (793.7 vs. 565.6 pg/mL, MWU test *p* = 0.96). However, differences between acute and convalescent phases were not statistically significant (WSR test *p* = 0.23), with convalescent-phase data missing for 12 patients.

### 3.3. Comparison of Plasma ANGPTL4 Concentrations Between Severe and Non-Severe Dengue Patients

**Figure 1:**
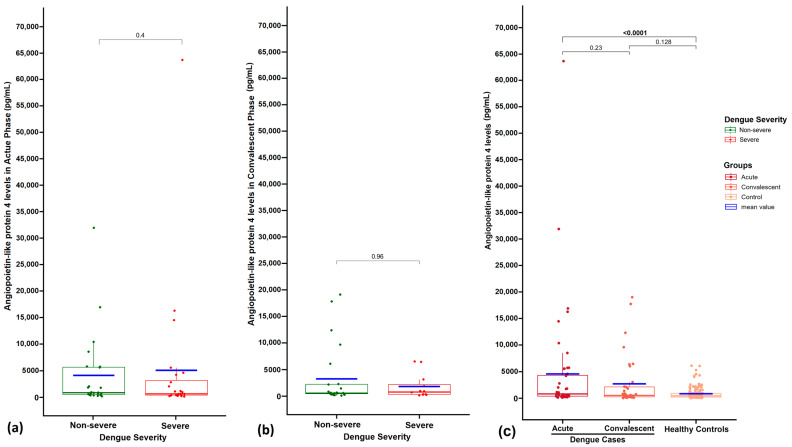
Plasma ANGPTL4 concentrations (pg/mL) between (**a**) severe and non-severe dengue patients in the acute phase; (**b**) severe and non-severe dengue patients in the convalescent phase; (**c**) dengue patients (acute and convalescent phases) and healthy control subjects. The *p*-values are indicated.

Table 2 compares ANGPTL4 concentrations between severe and non-severe dengue patients during the acute and convalescent phases of infection. In the acute phase, severe dengue cases had a median ANGPTL4 concentration of 677.4 pg/mL (IQR: 416.4 to 3206.1), while non-severe cases had a median of 909.1 pg/mL (IQR: 569.9 to 5679.0). However, this difference was not statistically significant (*p* = 0.4). During the convalescent phase, severe cases exhibited a median ANGPTL4 concentration of 793.7 pg/mL (IQR: 309.1 to 2187.6), compared to 565.6 pg/mL (IQR: 414.5 to 2191.8) in non-severe cases, albeit with no significant difference (*p* = 0.96). Within-group comparisons between acute and convalescent phases revealed no significant change in ANGPTL4 levels for severe cases (*p* = 0.176), albeit approaching statistical significance for non-severe cases (*p* = 0.056). These results suggest that ANGPTL4 concentrations do not significantly differ between severe and non-severe dengue cases in the acute or convalescent phases of infection (Figure 1).

The statistical test results are as follows:

o Severe dengue vs. non-severe dengue in acute phase: *p* = 0.4.
o Severe dengue vs. non-severe dengue in convalescent phase: *p* = 0.96.
o Acute phase vs. convalescent phase in severe dengue: *p* = 0.176.
o **Acute phase vs. convalescent phase in non-severe dengue: *p* = 0.056.**
o Acute phase vs. convalescent phase in dengue cases: *p* = 0.23.
o **Dengue cases in acute phase vs. healthy controls: *p* < 0.001.**
o Dengue cases in convalescent phase vs. healthy controls: *p* = 0.128.

### 3.4. Comparison of Plasma ANGPTL4 Concentrations Between Dengue Patients and Healthy Control Subjects

Table 2 shows ANGPTL4 concentrations (pg/mL) for dengue patients compared to healthy controls. In the acute phase of dengue (*n* = 48), the median concentration is 870.6 pg/mL and the mean is 4634.3 pg/mL, i.e., about five times higher than the mean of healthy controls (907.4 pg/mL). In the convalescent phase of dengue (*n* = 36), the median is 581.5 pg/mL and the mean is 2768 pg/mL, i.e., around three times higher than healthy controls. In dengue patients, acute phase ANGPTL4 levels are highly significantly elevated compared to healthy controls (*p* < 0.0001) while convalescent phase levels are higher but not statistically significant (*p* = 0.128) (Figure 1).

### 3.5. Association of Plasma ANGPTL4 Concentration with Plasma Leakage During the Acute Phase

**Figure 2:**
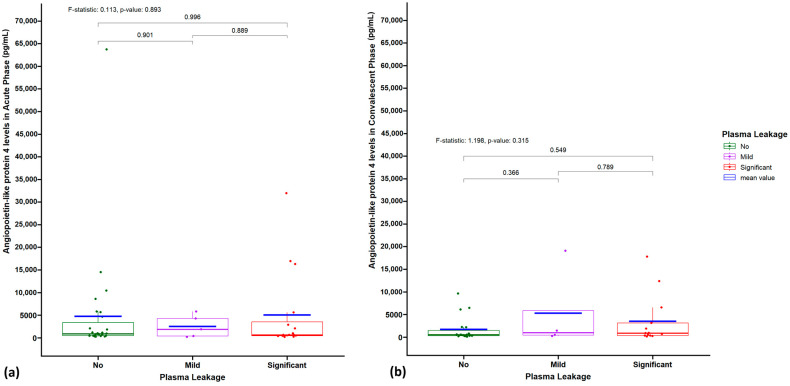
Plasma ANGPTL4 concentration (pg/mL) with extent of plasma leakage (**a**) in the acute phase and (**b**) in the convalescent phase of dengue virus infection. The *p*-values are indicated.

The association between plasma ANGPTL4 concentration and plasma leakage was analyzed in 48 dengue patients during the acute phase (Table 3). Of these, 27 patients had no plasma leakage, 5 had mild leakage, and 16 had significant leakage. The median ANGPTL4 levels were 897.1 pg/mL, 1870.5 pg/mL, and 649.9 pg/mL in the no, mild, and significant leakage groups, respectively. When ANGPTL4 levels were categorized into tertiles (low: 175.2 to 500.0; medium: 500.1 to 2000.0; high: 2000.1 to 63,737.6 pg/mL), the distribution across plasma leakage groups showed no significant differences (*p* = 0.829). Similarly, the Kruskal–Wallis test for comparing continuous ANGPTL4 values across plasma leakage groups showed no significant difference (*p* = 0.893) (Figure 2). These findings suggest that plasma ANGPTL4 concentration during the acute phase is not significantly associated with the severity of plasma leakage in dengue patients.

### 3.6. Association of Plasma ANGPTL4 Concentration with Severe Dengue During the Acute Phase

Table 4 presents the association between plasma ANGPTL4 concentrations during the acute phase and dengue severity. ANGPTL4 levels were categorized into tertiles and were analyzed as a continuous variable (per doubling). After adjusting for baseline demographics, plasma leakage, and comorbidities, a trend toward increased severity with higher ANGPTL4 levels was observed, albeit not reaching statistical significance. Compared to the lowest tertile (175.2 to 500.0 pg/mL), the medium tertile (500.1 to 2000.0 pg/mL) had an adjusted odds ratio (aOR) of 0.36 (95%CI: 0.08–1.65, *p* = 0.190) for severe dengue, while the highest tertile (2000.1 to 63,737.6 pg/mL) had an aOR of 0.57 (95%CI: 0.13–2.49, *p* = 0.456). Notably, the upper bounds of the confidence intervals revealed an increasing trend across tertiles, suggesting a potential association between higher ANGPTL4 levels and greater dengue severity. When analyzed as a continuous variable, each doubling of the ANGPTL4 concentration was associated with an aOR of 1.18 (95%CI: 0.95–1.47, *p* = 0.139) for severe dengue, further supporting this trend. Although not statistically significant, these trends warrant further investigations into the potential relationship between ANGPTL4 and dengue severity.

### 3.7. Clinical Characteristics of Dengue Patients with Very High Plasma ANGPTL4 Concentrations

We conducted further detailed analysis of the clinical and laboratory parameters of 11 patients who were all above the mean ANGPTL4 levels of 5122.3 pg/mL for acute samples of severe dengue patients (Supplementary Table S1). Among these eleven patients, four (36.4%) progressed to severe dengue according to the WHO 2009 criteria. Of these four, two had evidence of severe plasma leakage, one had severe bleeding, and the other had severe liver damage. Of note, four out of eleven patients (36.4%) with very high ANGPTL4 levels showed significant plasma leakage, while two (18.2%) had mild leakage. These findings, while descriptive, highlight a trend toward more severe dengue manifestations in patients with very high ANGPTL4 levels. Out of eleven patients, there were four (36.4%) individuals with severe dengue; four (36.4%) with dengue hemorrhagic fever and warning signs; two (18.2%) with dengue and thrombocytopenia; and only one (9.1%) with classical dengue fever. These disease severity patterns among a significant proportion of patients with very high ANGPTL4 levels suggest the potential role of ANGPTL4 and its utility as an early biomarker of dengue disease progression and warrant future detailed studies.

## 4. Discussion

Severe dengue can manifest as severe bleeding, organ damage, and plasma leakage due to the disruption of vascular integrity, leading to hypotension, shock, and circulatory collapse. This vascular dysfunction can result from both direct viral effects or immune-mediated responses [25]. To investigate the role of ANGPTL4 in dengue and its severity, we utilized plasma samples of the CODEN cohort to examine ANGPTL4 levels in 48 dengue patients (24 severe and 24 non-severe cases) during acute and convalescent phases of dengue. While no statistically significant differences in ANGPTL4 levels were found between severe and non-severe cases in acute and convalescent phases, our results suggest a trend toward increased severity with higher acute phase ANGPTL4 levels. Furthermore, plasma ANGPTL4 was significantly elevated in acute dengue (mean 4634.3 pg/mL) versus healthy controls (mean 907.4 pg/mL), with declining levels in the convalescent phase. Notably, 8 out of 11 patients (72.72%) with high ANGPTL4 levels (above the mean level of 5122.3 pg/mL) developed severe dengue or dengue hemorrhagic fever with warning signs, suggesting a potential association between plasma ANGPTL4 and dengue severity, particularly in relation to vascular leakage.

The ANGPTL4 continuous level analysis revealed a trend toward an increased probability of severe dengue with higher plasma ANGPTL4 (aOR = 1.18, 95% CI: 0.95–1.47), albeit statistically non-significant (*p* = 0.139). This is congruent with previous studies that have implicated ANGPTL4 in enhanced vascular permeability [15,19,20]. However, the tertile analysis presented a more complex picture: the more severe cases (10 out of 24) were observed in the lowest ANGPTL4 tertile (see Table 4). These findings suggest a complex relationship between plasma ANGPTL4 and dengue severity. ANGPTL4 undergoes proteolytic cleavage during inflammatory conditions, with the cleaved form binding to endothelial targets [26]. Increased cleavage and endothelial binding in severe dengue may lead to lower circulating ANGPTL4 levels despite higher overall activity. Additionally, ANGPTL4 expression may also undergo negative feedback regulation once certain thresholds are reached, as observed in ANGPTL3 [27]. The rising ANGPTL4 levels exhibit a concentration-dependent effect on vascular permeability [15,28], potentially contributing to the vascular leakage seen in severe dengue [3,29]. Furthermore, ANGPTL4 may interact with other inflammatory mediators as demonstrated in obesity-related breast cancer [30], e.g., by the upregulation of IL-1β in inflammatory conditions.

Li et al. [19] showed that an influenza virus infection can directly upregulate ANGPTL4 expression via a mechanism mediated by STAT3 and interleukin 6 (IL-6). STAT3 can be activated by IL-6 and interferon-gamma (IFN-γ) by viral infection [31]. Significantly higher levels of serum IL-6 and IFN-γ are observed in severe dengue patients, and elevated IL-6 levels are associated with dengue mortality [32,33,34]. Furthermore, IL-6 levels rise earlier in secondary dengue infections compared to primary dengue infections [35]. Evidence is accumulating that there is a complex interplay between IL-6 signaling and the vascular endothelium in disorders characterized by cytokine storms such as dengue [36]. In cells infected with the dengue virus, STAT3 is upregulated and activated by phosphorylation, and STAT3 acts as a crucial pro-viral factor for dengue virus replication [37]. Another proposed molecular mechanism of ANGPTL4 is its ability to activate the NF-κB inflammatory pathway and affect M1 macrophage polarization and pyroptosis in sepsis-related acute lung injury. This is noteworthy given that NF-κB serves as a critical transcriptional regulator of inflammation-related genes [38].

The role of ANGPTL4 in angiogenesis and vascular leakiness remains controversial. One study reported that ANGPTL4 prevents permeability and preserves vessel integrity, primarily in cancer [39]. Another study reported that ANGPTL4 counteracts the loss of vascular integrity in a murine transient ischemic stroke model [40]. ANGPTL4 can potentiate vascular disruption and leakage by the interaction of the C-terminal domain (C-ANGPTL4) with integrin α5β1, VE-cadherin, and claudin-5 [15]. ANGPTL4 is also required for maintaining endothelial cells, which is vital for vascular permeability and angiogenesis [41]. Bhatraju et al. [17] also demonstrated the close link between C-ANGPTL4 concentrations and COVID-19 clinical outcomes. In patients with acute respiratory distress syndrome, serum ANGPTL4 levels were about 5.7-fold higher than in control subjects (*p* < 0.0001) [16]. A study on community-acquired pneumonia revealed that ANGPTL4 levels were about 3.2 times higher in patients with severe pneumonia than those with non-severe disease (median of 1441.70 pg/mL versus 453.53 pg/mL, *p* < 0.001) [42]. The N-terminal and C-terminal fragments of ANGPTL4 mediate distinct roles in vascular permeability and angiogenesis, underscoring the need to differentiate their functions in a context- and tissue-dependent manner [43]. Our study did not distinguish between the C-ANGPTL4 and N-ANGPTL4 fragments—this limitation does not address the specific contributions of these fragments to dengue severity and vascular leakage.

While therapeutics targeting endothelial pathways in dengue infection are currently lacking, ANGPTL4 has emerged as a protein of interest in vascular permeability and endothelial function. In animal models, ANGPTL4 has shown utility in preserving vascular integrity and reducing infarct size in ischemic heart disease [44] and in modulating vascular permeability in diabetic macular edema [28]. As a regulator of lipoprotein lipase, ANGPTL4 modulation impacts lipid homeostasis, thus rendering it a promising therapeutic target for cardiovascular disorders [45]. The involvement of ANGPTL4 in tumorigenesis, stem cell, and vascular functions further expands its therapeutic potential [11]. However, further investigations are needed to elucidate the regulatory mechanisms and pathways of ANGPTL4 in order to develop targeted therapies for various vascular pathophysiologic conditions and to align these molecular insights with clinical applications [46].

This study has several strengths. Firstly, we utilized samples from the CODEN, a well-characterized prospective cohort of dengue patients in Singapore. This cohort provided a robust foundation for our investigation, ensuring the reliability and clinical relevance of our findings. Secondly, our study compared plasma ANGPTL4 concentrations in dengue patients versus healthy controls, thus facilitating meaningful comparisons and establishing baseline levels. Thirdly, the long-term stability of ANGPTL4 at −80 °C storage conditions, as demonstrated in previous studies [47], ensures the reliability of our measurements using stored samples. Lastly, our use of multivariable models adjusted for demographics and comorbidities suggested that ANGPTL4 has an independent association with dengue severity, strengthening the potential significance of our findings.

However, this study also has some limitations. We did not examine the ANGPTL4 association with specific DENV serotypes, which may provide insights into virus serotype-specific pathogenesis [25]. Our ANGPTL4 measurement did not differentiate between the C-terminal and N-terminal ANGPTL4 fragments, which mediate distinct biological functions. Additionally, our analysis lacked plasma samples at multiple time-points throughout the acute and convalescent dengue phases, limiting our ability to determine the temporal trends of ANGPTL4 levels during the progression from mild to moderate to severe dengue.

ELISA is most widely used to measure biomarkers in blood, including C-reactive protein (CRP), which is a biomarker of inflammatory conditions including dengue [48]. Enzyme immunoassays are well established and highly automated in many hospital settings and are offered at a reasonable cost (e.g., currently about USD 22 for a CRP test in Singapore). Innovative strategies are being developed to further reduce the cost of ELISA to cheaper and rapid point-of-care (POC) tests such as the COVID-19 antigen rapid test (which currently costs about USD 4 per test in Singapore). Technological advances to render such POC tests more rapid, accessible, and cost effective include paper-based lateral flow immunoassays and biosensors [49,50]. Potential blood biomarkers of vascular leakage in dengue may also be complemented by other parameters such as vascular nitric oxide bioavailability measured by reactive hyperemia peripheral arterial tonometry [51].

## 5. Conclusions

In conclusion, this study revealed that plasma ANGPTL4 levels were significantly elevated in dengue patients compared to healthy subjects. This suggests that ANGPTL4 may be relevant as a biomarker or therapeutic target, which warrants further investigations into the relationship between ANGPTL4 levels and dengue severity. For better statistical power, larger prospective studies with an analysis of serial samples at multiple time-points are necessary to elucidate ANGPTL4 expression trends throughout dengue illness, DHF and DSS, including in pediatric cohorts where vascular permeability is a key concern. Examining ANGPTL4 in relation to other related angiopoietin proteins and cytokines previously implicated in dengue and other flaviviral diseases may also provide important insights into dengue pathogenesis [52,53,54,55,56]. Future research may help to clarify the role of ANGPTL4 especially in severe dengue manifestations.

## Figures and Tables

**Table 1:** Clinical and laboratory characteristics of selected dengue patients.

Characteristics	Overall (*n* = 48)	Severe Dengue		*p*-Value
		No (*n* = 24)	Yes (*n* = 24)	
**Age (years)**	**45.7 (15.4)**	42.7 (15.3)	48.8 (15.1)	0.2 *
**Gender**				**0.043** ^§^
Male	25 (52.08%)	16 (66.67%)	9 (37.50%)	
Female	23 (47.92%)	8 (33.33%)	15 (62.50%)	
**Ethnicity**				>0.9 ^§§^
Chinese	38 (79.17%)	19 (79.17%)	19 (79.17%)	
Indian	1 (2.08%)	1 (4.17%)	0 (0%)	
Malay	1 (2.08%)	0 (0%)	1 (4.17%)	
Others	8 (16.67%)	4 (16.67%)	4 (16.67%)	
**In/Outpatient**				**0.004** ^§§^
Inpatient	40 (83.33%)	16 (66.67%)	24 (100%)	
Outpatient	8 (16.67%)	8 (33.33%)	0 (0%)	
**Comorbidity**				0.712 ^§^
No	39 (81.25%)	20 (83.33%)	19 (79.17%)	
Any	9 (18.75%)	4 (16.67%)	5 (20.83%)	
**DHF**				**0.033** ^§^
Yes	10 (20.83%)	2 (8.33%)	8 (33.33%)	
No	38 (79.17%)	22 (91.67%)	16 (66.67%)	
**Plasma leakage**				**0.005** ^§§^
Significant	16 (33.33%)	3 (12.50%)	13 (54.17%)	
Mild	5 (10.42%)	4 (16.67%)	1 (4.17%)	
No	27 (56.25%)	17 (70.83%)	10 (41.67%)	
**ANGPTL4 concentration (pg/mL) in acute phase**				0.4 ^⁑^
Mean (SD)	4634.3 (10,525.3)	4146.3 (7209.1)	5122.3 (13,187.7)	
Median (IQR)	870.6 (441.7, 4368.4)	909.1 (569.9, 5679.0)	677.4 (416.4, 3206.1)	
**ANGPTL4 concentration (pg/mL) in convalescent phase**				0.96 ^⁑^
Mean (SD)	2768.0 (4789.3)	3245.3 (5618.2)	1813.5 (2344.0)	
Median (IQR)	581.5 (315.3, 2191.8)	565.6 (414.5, 2191.8)	793.7 (309.1, 2187.6)	
Missing	12	0	12	

Data presented are mean (standard deviation) or frequency (percentage) where appropriate. ANGPTL4: angiopoietin-like 4 protein; DHF: dengue hemorrhagic fever; IQR: interquartile range; SD: standard deviation; * Welch two-sample *t*-test; ^⁑^ Wilcoxon rank sum exact test; ^§^ Pearson’s chi-squared test; ^§§^ Fisher’s exact test. The *p*-values in bold font indicate statistical significance.

**Table 2:** Comparison of plasma ANGPTL4 concentrations between severe and non-severe dengue cases during acute and convalescent phases and healthy controls.

Samples	Plasma ANGPTL4 Concentration (pg/mL)		
	*n*	Median (IQR)	Mean ± SD
**Acute phase**	**48**	**870.6 (441.7, 4368.4)**	**4634.3 ± 10,525.3**
Severe dengue	24	677.4 (416.4, 3206.1)	5122.3 ± 13,187.7
Non-severe dengue	24	909.1 (569.9, 5679.0)	4146.3 ± 7209.1
**Convalescent phase**	**36**	**581.5 (315.3, 2191.8)**	**2768.0 ± 4789.3**
Severe dengue	12	793.7 (309.1, 2187.6)	1813.5 ± 2344.0
Non-severe dengue	24	565.6 (414.5, 2191.8)	3245.3 ± 5618.2
**Normal control subjects**	**152**	**504.4 (272.9, 978.2)**	**907.4 ± 1113.7**

ANGPTL4: angiopoietin-like 4 protein; IQR: interquartile range; SD: standard deviation; *n*: sample size. Mann–Whitney U test was used for between-group comparisons due to the non-normal distribution of data. Wilcoxon signed-rank test was used for within-group comparisons (acute versus convalescent) due to the paired nature of data and non-normal distribution.

**Table 3:** Association of plasma ANGPTL4 concentration with extent of plasma leakage in the acute phase of dengue.

Plasma ANGPTL4 Concentration (pg/mL) in Acute Phase	Overall (*n* = 48)	Plasma Leakage			*p*-Value
		No (*n* = 27)	Mild (*n* = 5)	Significant (*n* = 16)	
**Median (IQR)**	870.6 (441.7, 4368.4)	897.1 (474.3, 3372.2)	1870.5 (386.8, 4274.4)	649.9 (429.7, 3538.4)	0.893 ^§^
**Mean ± SD**	4634.3 ± 10,525.3	4781.1 ± 12,313.6	2506.0 ± 2460.2	5051.8 ± 8997.5	
**Tertile 1** (low): 175.2–500.0	16 (33.33%)	8 (29.63%)	2 (40.00%)	6 (37.50%)	0.829 ^§§^
**Tertile 2** (medium): 500.1–2000.0	16 (33.33%)	11 (40.74%)	1 (20.00%)	4 (25.00%)	
**Tertile 3** (high): 2000.1–63,737.6	16 (33.33%)	8 (29.63%)	2 (40.00%)	6 (37.50%)	

ANGPTL-4: angiopoietin-like 4 protein; IQR: interquartile range; SD: standard deviation; *n*: sample size; ^§^ Kruskal–Wallis rank sum test; ^§§^ Fisher’s exact test.

**Table 4:** Association of plasma ANGPTL4 concentration with severe dengue in acute phase.

Plasma ANGPTL4 Concentration (pg/mL) (Acute Phase)	*n * at Risk	*n* Events	Baseline Demographic Adjusted ^a^		Multivariable Adjusted ^b^	
			^a^ OR (95%CI)	*p*-Value	^a^ OR (95%CI)	*p*-Value
**Tertile 1 (low):** **175.2–500.0**	16	10	1.0 (Reference)	-	1.0 (Reference)	-
**Tertile 2 (medium):** 500.1–2000.0	16	6	0.41 (0.10–1.60)	0.200	0.36 (0.08–1.65)	0.190
**Tertile 3 (high):** 2000.1–63,737.6	16	8	0.66 (0.18–2.40)	0.527	0.57 (0.13–2.49)	0.456
**Per doubling: log2(ANGPTL4)**	48	24	1.15 (0.96–1.38)	0.137	1.18 (0.95–1.47)	0.139

ANGPTL4: angiopoietin-like 4 protein; CI: confidence interval; OR: odds ratio. ^a^ Baseline demographic adjusted: age and gender. ^b^ Multivariable adjusted: baseline demographic covariates, plasma leakage, and comorbidity. Multivariable logistic regression was adjusted to test an association between ANGPTL4 tertiles or ANGPTL4 continuous (doubling) with severe dengue outcome.

## Data Availability

The raw data supporting the conclusions of this article will be made available by the authors on request.

## Supplementary Materials

The following supporting information can be downloaded at https://www.mdpi.com/article/10.3390/v17020226/s1: Supplementary Table S1: Clinical characteristics of dengue patients with very high plasma ANGPTL4 concentration (pg/mL). Supplementary Figure S1: Comparison of ANGPTL4 concentrations between the acute phase and convalescent phase in patients with non-severe and severe dengue.
