# Supplementary material for: Elevated Plasma Angiopoietin-like 4 Protein Levels in Adult Patients with Dengue
Source: Viruses. 2025 Feb 6;17(2):226. doi: 10.3390/v17020226 (PMC11861331; doi:10.3390/v17020226)
Supplement: Supplementary file 1 [file viruses-17-00226-s001.zip › Suppl Table S1 & Figure S1 Dengue ANGPTL4 Feb2025.pdf]

Supplementary Table S1. Clinical characteristics of dengue patients with very high plasma ANGPTL4 concentration (pg/mL).

Characteristics	Patient 1	Patient 2	Patient 3	Patient 4	Patient 5	Patient 6	Patient 7	Patient 8	Patient 9	Patient 10	Patient 11
**Severe Dengue (2009)**	**Yes (Menorrhagia)**	**Yes (Blood Transfusion)**	**Yes (High ALT, AST)**	**Yes (Severe Plasma Leakage AND Shock)**	No	No	No	No	No	No	No
1. Significant/Severe Bleeding (2009)	**Yes (Menorrhagia)**	**Yes (Blood Transfusion)**	No	No	No	No	No	No	No	No	No
2. Severe organ involvement	No	No	**Yes (High ALT, AST)**	No	No	No	No	No	No	No	No
3. Severe Plasma Leakage (2009) AND Shock (2009)	No (only Hypotension for Age SBP<=90)	No (only Severe Plasma Leakage)	No	**Yes (Both)**	No (only Severe Plasma Leakage)	No (only Severe Plasma Leakage)	No (only Hypotension for Age SBP<=90)	No (only Hypotension for Age SBP<=90)	No	No	No
3a. Severe Plasma Leakage (2009)	No	**Yes (HCT Change >=20%)**	No	**Yes (HCT Change >20% AND Pleural Effusion & Ascites both clinical and radiological)**	**Yes (HCT Change >=20%)**	**Yes (HCT Change >20% AND Radiological Pleural Effusion)**	No	No	No (only Hypoproteinemia)	No	No
3b. Shock (2009)	No	No	No	**Yes**	No	No	No	No	No	No	No

**Plasma Leakage (No, Mild, Significant)**	No	**Significant**	No	**Significant**	**Significant**	**Significant**	No	No	Mild	No	No
**ANGPTL4 (Acute)**	63737.6	16318.5	14519.1	5603.5	31971.1	16950	10417.2	5643.9	5801.1	8586.9	5784.3
**ANGPTL4 (Convalescent)**	NA	6534.6	6442.7	1880.8	12357.6	17791.6	9639.4	6098.2	19074.1	281.0	2173.9
**Age**	26	24	52	49	22	65	32	45	39	82	22
**Gender**	Female	Female	Female	Male	Male	Female	Male	Male	Male	Male	Female
**Patient Type**	Outpatient/ Inpatient	Outpatient / Inpatient	Inpatient	Inpatient	Inpatient	Inpatient	Inpatient	Inpatient	Outpatient	Outpatient/ Inpatient	Outpatient
**Platelets**	**16 × 10^9/L**	117 × 10^9/L	119 × 10^9/L	**4 × 10^9/L**	**44 × 10^9/L**	**30 × 10^9/L**	**17 × 10^9/L**	**41 × 10^9/L**	**92 × 10^9/L**	**17 × 10^9/L**	103 × 10^9/L
**Neutrophils**	0.5 × 10^9/L	1.16 × 10^9/L	1.07 × 10^9/L	1.74 × 10^9/L	0.53 × 10^9/L	0.37 × 10^9/L	0.92 × 10^9/L	0.6 × 10^9/L	**27 × 10^9/L**	1.35 × 10^9/L	0.38 × 10^9/L
**Outcomes**	**Severe Dengue**	**Severe Dengue**	**Severe Dengue**	**Severe Dengue**	Dengue with **Warning Signs**	Dengue with **Warning Signs**	Dengue with **Warning Signs**	Dengue with **Warning Signs**	Dengue with **thrombocyte-penia** and **neutrophilia**	Dengue with **thrombocyto-penia**	Dengue Fever

ANGTPL4: Angiopoietin-like 4 protein; AST: Aspartate transaminase; ALT: Alanine transaminase; HCT: Hematocrit

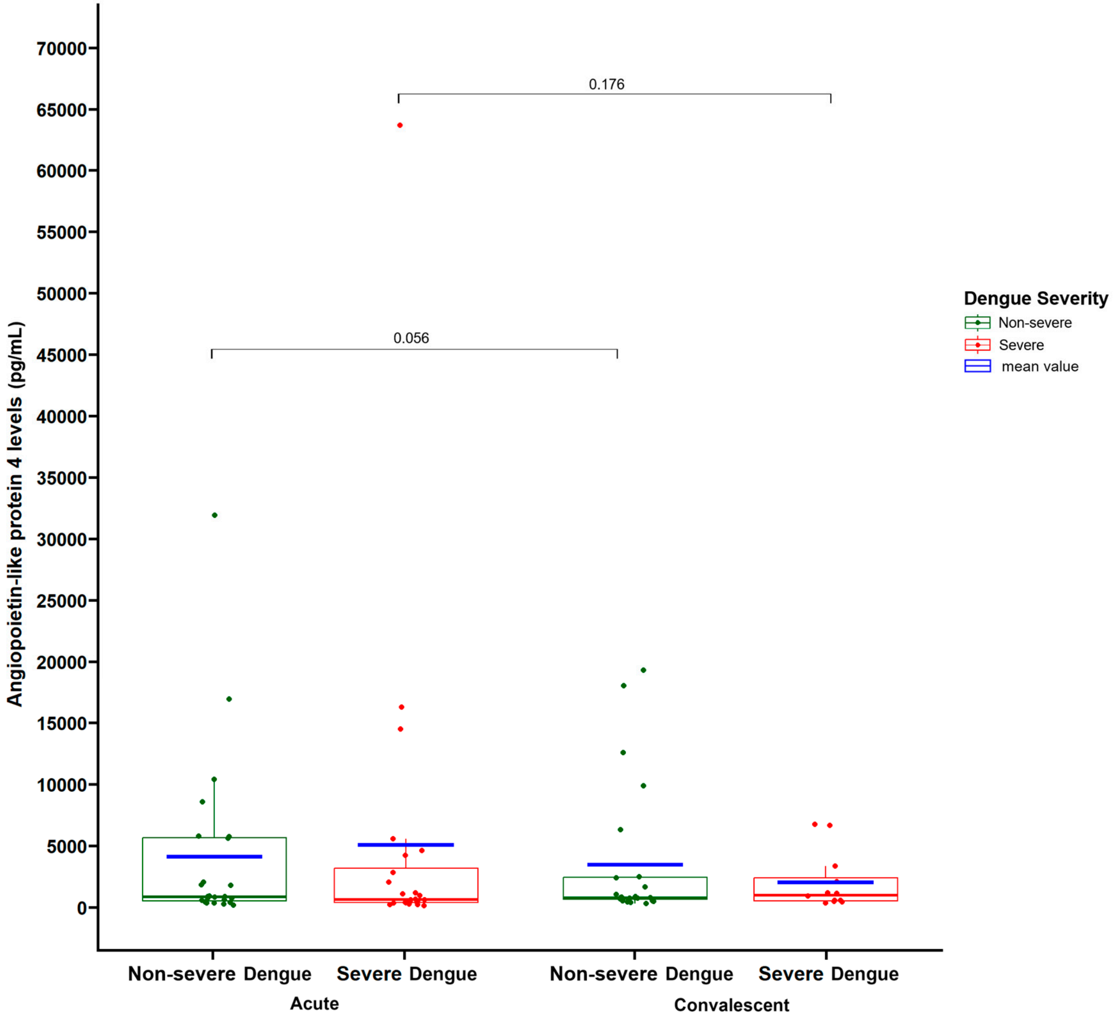

Supplementary Figure S1. Comparison of ANGPTL4 concentrations between the acute phase and convalescent phase in patients with non-severe and severe dengue. The *p*-values are indicated.
